# Aesthetic Evaluation of the Nasolabial Region in Children with Unilateral Cleft Lip and Palate Comparing Expert versus Nonexperience Health Professionals

**DOI:** 10.1155/2014/460106

**Published:** 2014-07-13

**Authors:** Tatiana Saito Paiva, Marcia Andre, Wellingson Silva Paiva, Beatriz Silva Camara Mattos

**Affiliations:** Department of Maxillofacial Surgery, Prosthodontics, and Traumatology, Dentistry School, University of Sao Paulo, Alves Guimarães Street 470, 05410000 Sao Paulo, SP, Brazil

## Abstract

Esthetic evaluation of cleft lip and palate rehabilitation outcomes may assist in the determination of new surgical interventions and aid in reevaluation of treatment protocols. Our objective was to compare esthetics assessments of the nasolabial region in children with a unilateral cleft lip and palate between healthcare professionals who were experienced in the treatment of cleft lip and palate and those who were inexperienced. The study group included 55 patients between 6 and 12 years of age who had already undergone primary reconstructive surgery for unilateral cleft lip. Standardized digital photographs were obtained, and the esthetic features of the nose, lip, and nasolabial region were evaluated. We used only cropped photographic images in the assessments of healthcare professionals with and without experience in cleft lip and palate. Interrater analysis revealed highly reliable assessments made by both the experienced and inexperienced professionals. There was no statistically significant difference in the esthetic attractiveness of the lip and nose between the experienced and inexperienced professionals. Compared with the inexperienced professionals, the experienced professional evaluators showed higher satisfaction with the esthetic appearance of the nasolabial region; however, no difference was observed in the analysis of the lip or nose alone.

## 1. Introduction

Congenital and acquired anatomic abnormalities that interfere with the anatomy of the face can cause cognitive and psychological sequelae. In patients with cleft lip and palate, the consequences can be negative psychosocial changes ranging from low self-esteem to risk of social isolation. Therefore, the esthetic evaluation of individuals with cleft lip and palate is an important clinical indicator in the analysis of facial deformities and planning of surgical treatment [1]. The esthetic appearance of the nasolabial region is one of the most important tools to evaluate the success of treatment [2]. Different surgical intervention techniques are used for correction of cleft lip and palate, but the results are generally analyzed subjectively and without standardization and depend on the particular perspective of the observer, which may be influenced by ethnic or cultural background as well as age, making interpretation difficult.

Both quantitative and qualitative methods of esthetic evaluation appear in the literature. Quantitative methods use anthropometric measurements of the facial soft tissue, obtained by manual or automatic measurement, intended to quantify the morphology of the lip and the nose and the degree of asymmetry [3, 4]. This approach is suitable for an analysis of asymmetries but neglects the harmony of the face [4]. Qualitative studies, with different designs and combinations of evaluators, use either ordinal or visual analog scales [5]. Although these methods entail a more subjective analysis of facial esthetics, they may provide the best reflection of the general population's perception of the patient's facial appearance. A method that has become quite popular over the last decade is the index developed by Asher-McDade et al. [6]. This index was used in the Eurocleft Study [7], and, subsequently, several other studies [8, 9] confirmed it to be a reliable and reproducible method of classification in the assessment of nasolabial appearance. However, considering the methodology of the analysis of facial attractiveness, it is important to determine whether the esthetic evaluation of professionals experienced in the treatment of cleft lip and palate correlates with the view of society in general [10].

In studies published so far, marked differences exist within the evaluator groups; for example, a group of experienced professionals could include only plastic surgeons or could include professionals from different fields, for example, orthodontists, psychologists, surgeons, and others. The lay group may also be diversified and include individuals unrelated to the health sector or even the cleft patient himself. To the best of our knowledge, however, no studies have compared professionals experienced in the treatment of cleft lip and palate with health professionals without that experience, who are often the first professional contact for these patients. In the present study, our objective was to determine whether there was a correlation between the esthetic assessments performed by health professionals who are inexperienced and those who are experienced in the treatment of cleft lip and palate.

## 2. Methods

### 2.1. Setting

We performed a prospective study from September 2012 to August 2013. Data were collected from 55 cleft lip and palate patients who were selected from the Maxillofacial Prosthodontics Clinics of the University of São Paulo School of Dentistry. Patients between 6 and 12 (mean 1.98 ± 8.41) years of age were selected, regardless of race or gender, with unilateral cleft lip and palate. Patients included in the study had undergone reconstructive surgery of the lip between 3 and 6 months of age and palatoplasty between 18 and 24 months of age. In the total sample, 35 were boys and 20 were girls. Individuals who showed any anomaly characterized as syndromic or had already undergone bone graft surgery in the alveolar cleft region were excluded. The procedures in this study were previously approved by the School of Dentistry Ethics Committee, University of São Paulo, and were in accordance with the principles of the Declaration of Helsinki.

### 2.2. Evaluators

Evaluators were healthcare professionals divided into 2 groups: those who were experienced and those who were inexperienced in the treatment of cleft lip and palate. The 4 experienced professionals included a plastic surgeon, 2 orthodontists, and a pediatric dentist. The 6 inexperienced professionals included 2 doctors (a pediatrician and a general practitioner) and 4 dentists.

### 2.3. Evaluation Parameters

**Figure 1:**
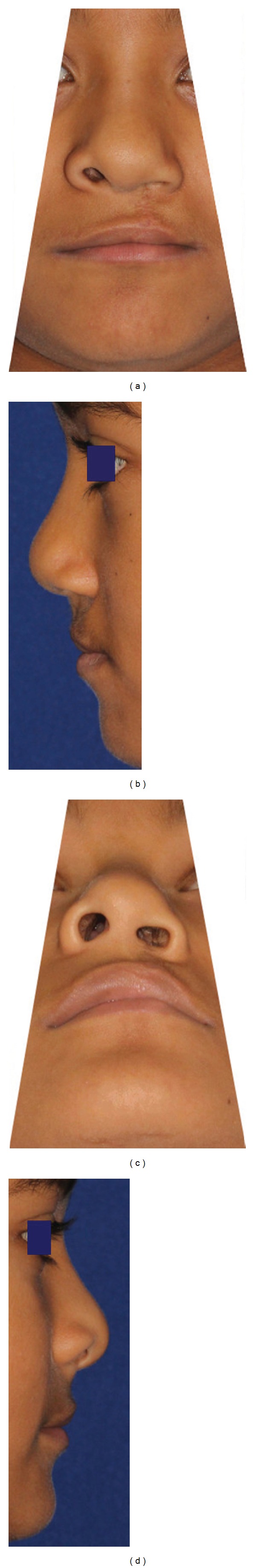
Representative standardized photos taken of each patient. (a) Front view. (b) Left lateral view. (c) Submental oblique view. (d) Right lateral view.

For esthetic evaluation of the nasolabial region of the patients, standardized digital photographs highlighting only the nasolabial region, total face, right profile, left profile, and submental oblique view were used (Figure 1). For each patient, 4 photographs were taken. The photo was standardized according to the recommendations by the Institute of Medical Illustrators [11]. The blue color was used as a photographic backdrop for better contrast with the skin color and to minimize the occurrence of shadows [12].

The lip and nose were evaluated individually, and the nasolabial region was evaluated as a whole. The esthetic aspects analyzed were symmetry and lip volume, continuity of the vermilion of the upper lip, scar, symmetry of the nose tip and columella, alar base insertion, nasolabial angle, and maxillomandibular relationship.

We used the classification method proposed by Asher-McDade et al. [6], which consists of a 5-point scale where 1 represents very good appearance and 5 represent very poor appearance.

We first calculated the interrater analysis separately within each group of evaluators and subsequently evaluated the differences in satisfaction with the appearance of the lip, nose, and nasolabial region between the rater groups.

### 2.4. Statistical Analyses

Statistical analyses were performed using SPSS (IBM, Chicago, IL). The Cronbach's alpha test statistic was used to analyze the degree of agreement between the ratings from the 2 groups of evaluators. The Wilcoxon signed-rank test was used to analyze the differences between the 2 groups in satisfaction with the appearance of the lip, nose, and nasolabial region. For both analyses, a *P* value < 0.05 was considered significant.

## 3. Results

The interrater analyses indicated a high degree of agreement in the ratings given by the 4 experienced professionals (Table 1) and the 6 inexperienced professionals (Table 2) regarding the esthetic of the lip, nose, and nasolabial region (all *P* values < 0.05). It can be inferred, a priori, that the data are internally consistent and that the sample shows a high degree of reliability.

Noting the differences in satisfaction with the appearance of the lip, nose, and nasolabial region among the experienced and inexperienced professional evaluators, we observed no statistically significant differences for lip and nose between the 2 groups of evaluators. However, for the nasolabial region, the experienced professionals' satisfaction rating was significantly higher than that of the inexperienced professionals (*P* = 0.002) (Table 3).

## 4. Discussion

In the present study, we evaluated the influence of the nasolabial region on facial esthetics in patients with unilateral cleft lip and palate. Our results showed high reliability and reproducibility in the assessment scale applied by healthcare professionals with and without experience in treating children with clefts. Sharma et al. [13] found that some techniques used to evaluate facial esthetics in cleft patients proved to be inefficient because of problems inherent in their design and methodology. According to Alley and Hildebrandt [14], however, human judgment can act as a reliable tool in the assessment of facial attractiveness. Qualitative methods are more commonly used than quantitative methods, but the ordinal scales for rating the esthetic results in patients with cleft palate vary widely, with evaluators using 4 [15], 5 [6, 8, 16, 17], 6 [18, 19], 7 [20], 9 [21], or 10 [15] evaluation gradations.

In the present study, we adopted the rating scale of Asher-McDade et al. [6] for analysis of standardized clinical photographs, and the high interrater agreement confirmed the applicability of this classical scale. The observed high reproducibility and reliability, in relation to the group of evaluators, are found in other studies [6, 10, 22]. However, Power and Matic [23], using the same scale as Asher-McDade et al. [6], reported that experienced surgeons showed poor agreement between their esthetic evaluations of lip, suggesting that divergence in relation to other published studies could reflect a personal bias when a surgeon judges postoperative results of another surgeon.

Nollet et al. [8] stated that the average results may be used if the coherence between the components of the individual groups is substantial. For this reason, in this comparative study between groups of experienced and inexperienced professional evaluators, we used the average of each group's esthetic analysis of the lip, nose, and nasolabial region.

In the present study, we found no differences in the esthetic analysis of lip and nose between professionals experienced and inexperienced in the treatment of cleft lip and palate; however, in assessing the nasolabial region as a whole, the experienced professionals showed higher satisfaction with the esthetic results than the inexperienced professionals.

Some authors [10, 22, 24, 25] also reported that experienced practitioners showed higher satisfaction with the appearance of the nasolabial region than lay assessors. However, there are studies that have reported opposite results, with the lay assessors giving higher attractiveness ratings than the experienced professionals [18, 26]. Furthermore, some studies have shown no differences in ratings between professionals and lay people [20, 27]. The inconsistencies of these findings could be a result of methodological differences, such as the diversity in the constitution and in the number of evaluators. Additionally, whether the nasolabial region is evaluated as a whole or the nose and lip structures evaluated individually and whether an initial calibration is performed between evaluators could also explain the discrepancies found in the literature.

## 5. Conclusion

The assessment of esthetic outcomes in patients with cleft lip and palate is complex. Experienced evaluators in the treatment of cleft patients showed higher satisfaction with the cosmetic appearance of the nasolabial region than the inexperienced health professionals, but for isolated analysis of the lip or nose there was no difference. Thus, future classification systems should be developed according to the principles of clinical applicability, enabling different health professionals, even those without experience with this pathology, to perform the esthetic evaluations with high reproducibility, even though in many areas these professionals are currently responsible only for the clinical care of these patients.

## Figures and Tables

**Table 1:** Cronbach's alpha test: verification of the degree of agreement among experienced evaluators for esthetic evaluation of the lip, nose, and nasolabial region.

Regions assessed	Experienced evaluators	Mean ± SD rating	Cronbach's alpha coefficient	*P*
Lip	1	2.64 ± 1.22	0.881	**<0.001**
	2	2.60 ± 0.99		
	3	2.84 ± 1.01		
	4	2.82 ± 1.12		

Nose	1	2.95 ± 1.21	0.888	**<0.001**
	2	3.07 ± 1.20		
	3	3.25 ± 0.89		
	4	3.27 ± 1.10		

Nasolabial region	1	2.80 ± 0.89	0.848	**<0.001**
	2	2.78 ± 0.74		
	3	3.02 ± 0.76		
	4	3.09 ± 0.73		

SD: standard deviation.

**Table 2:** Cronbach's alpha test: verification of the degree of agreement among inexperienced evaluators for esthetic evaluation of the lip, nose, and nasolabial region.

Regions assessed	Inexperienced evaluators	Mean ± SD rating	Cronbach's alpha coefficient	*P*
Lip	1	2.56 ± 1.12	0.878	**<0.001**
	2	2.67 ± 1.16		
	3	2.84 ± 1.20		
	4	2.55 ± 0.79		
	5	2.49 ± 0.98		
	6	3.44 ± 0.66		

Nose	1	2.29 ± 1.33	0.814	**<0.001**
	2	3.00 ± 1.32		
	3	3.38 ± 1.01		
	4	2.76 ± 0.69		
	5	2.75 ± 0.89		
	6	3.85 ± 0.41		

Nasolabial region	1	2.80 ± 0.83	0.796	**<0.001**
	2	3.13 ± 1.12		
	3	3.31 ± 1.07		
	4	2.98 ± 0.71		
	5	2.55 ± 0.77		
	6	3.67 ± 0.51		

SD: standard deviation.

**Table 3:** Wilcoxon signed-rank post hoc test: verification of the differences between experienced and inexperienced professional evaluators for the lip, nose, and nasolabial region.

Variable pairs	*N*	Mean rating	SD	Minimum	Maximum	25th percentile	50th percentile (median)	75th percentile	*P*
Lip experienced	55	2.72	0.94	1.00	4.75	2.00	2.75	3.25	0.458
Lip inexperienced	55	2.76	0.79	1.33	4.33	2.00	2.83	3.33	

Nose experienced	55	3.14	0.96	1.00	4.75	2.50	3.00	4.00	0.067
Nose inexperienced	55	3.01	0.72	1.50	4.50	2.50	2.83	3.67	

NR experienced	55	2.92	0.65	1.00	4.25	2.50	3.00	3.25	**0.002**
NR inexperienced	55	3.07	0.60	1.83	4.33	2.67	3.17	3.50	

NR: nasolabial region; SD: standard deviation.
